# The Fear to Move in a Crowded Environment. Poor Spatial Memory Related to Agoraphobic Disorder

**DOI:** 10.3390/brainsci11060796

**Published:** 2021-06-16

**Authors:** Micaela Maria Zucchelli, Laura Piccardi, Raffaella Nori

**Affiliations:** 1 Department of Psychology, University of Bologna, 40127 Bologna, Italy; micaela.zucchelli3@unibo.it; 2 Department of Psychology Sapienza, University of Rome, 00185 Rome, Italy; laura.piccardi@uniroma1.it; 3 Cognitive and Motor Rehabilitation and Neuroimaging Unit, IRCCS Fondazione Santa Lucia, 00185 Rome, Italy

**Keywords:** agoraphobia, virtual environment, visuo-spatial working memory, crowded environment, early diagnosis

## Abstract

Individuals with agoraphobia exhibit impaired exploratory activity when navigating unfamiliar environments. However, no studies have investigated the contribution of visuospatial working memory (VSWM) in these individuals’ ability to acquire and process spatial information while considering the use of egocentric and allocentric coordinates or environments with or without people. A total of 106 individuals (53 with agoraphobia and 53 controls) navigated in a virtual square to acquire spatial information that included the recognition of landmarks and the relationship between landmarks and themselves (egocentric coordinates) and independent of themselves (allocentric coordinates). Half of the participants in both groups navigated in a square without people, and half navigated in a crowded square. They completed a VSWM test in addition to tasks measuring landmark recognition and egocentric and allocentric judgements concerning the explored square. The results showed that individuals with agoraphobia had reduced working memory only when active processing of spatial elements was required, suggesting that they exhibit spatial difficulties particularly in complex spatial tasks requiring them to process information simultaneously. Specifically, VSWM deficits mediated the relationship between agoraphobia and performance in the allocentric judgements. The results are discussed considering the theoretical background of agoraphobia in order to provide useful elements for the early diagnosis of this disorder.

## 1. Introduction

“*There are too many people in this square, I do not see any escape routes ... I want to get out of here*!!!” exclaims a person with agoraphobia (AG). Typically, people with AG experience panic in situations where they may lose some control. For instance, people with AG may avoid public transportation because they are not the ones driving the vehicles or avoid other situations (both closed and open spaces) such as movie theatres, lifts or fields, lakes, and parking lots, as well as any areas where crowds gather. AG has long been classified in accordance with one of two main perspectives. One considers AG to be a severe subtype of panic disorder and a biological defect, characterized by recurring panic attacks [1]. The other considers AG within a cognitive-behavioural approach as a disease distinct from panic disorders [2]. Several recent empirical studies have interpreted AG as an independent disease, leading to its separation from panic disorders in the DSM 5 [3]. Accordingly, AG consists of a marked fear or anxiety in situations from which escape might be difficult. These situations, as described above, may include open and closed spaces or simply being outside the home and may possibly result in a panic attack. Patients with AG have significant restrictions in lifestyle, suffering from one of the most disabling of all phobias [4,5] because of the constant need to move through environments to reach everyday destinations. Environmental navigation is a complex process that involves acquiring information and locating oneself with respect to a landmark or to an absolute coordinate system [6]. Indeed, to orient yourself and reach a destination, you have to follow some steps: (a) determine self-location and estimate goal location; (b) select routes from the starting point to a target destination; (c) monitor the route; and (d) recognize the target [7,8]. This ability depends on the integration of numerous cognitive functions [9,10]. According to the “self-reference” model [11], there are two main systems involved in reaching a goal: (1) the “egocentric” (body-centred) frame of reference, which has a specific role in guiding movement in real time since spatial relations are continuously updated as a person moves through an environment; and (2) the “allocentric” (world-centred) frame of reference, which is based on a long-term memory representation of the environment (e.g., [8,12,13,14]). In other words, when individuals navigate through an environment, they memorize spatial locations by considering body-centred coordinates and using the body axes of front–back, right–left, and up–down (i.e., the egocentric system). On the other hand, when they code the spatial relations among objects in the environment and study a path on a map, they use an orientation reference system independent from their position (i.e., the allocentric system). One of the most studied cognitive processes underlying navigation is visuo-spatial working memory (VSWM), which is used for manipulating, updating, and monitoring visuo-spatial information. VSWM includes two different components, a passive component (visual cache) responsible for temporarily storing visual information and an active component (inner scribe) responsible for the processing and manipulation of spatial information [15]. The importance of VSWM in navigation has been extensively demonstrated [16] using different methods including real-world wayfinding [8] and focusing on healthy individuals [8,13,17], age-related changes [18], and individuals with cognitive decline [19]. Findings have consistently identified poor VSWM as responsible for unsuccessful navigation [20]. Prior studies have identified VSWM as a mediator between executive function and spatial tasks, as well as a predictor of written arithmetical skills in children [21]. Therefore, VSWM could be considered a cognitive process underlying different abilities that mediates the relationship among different cognitive processes [21]. The few studies [22] investigating navigation in patients with AG or panic disorder (PD) with AG have reported deficits due to an automatic attentional bias. Specifically, Kállai et al. [22] proposed that during a panic attack or in agoraphobia avoidance, individuals (1) feel anxiety and fear; (2) are so focused on their physical reactions that they fail to perceive environmental cues and to create the environmental cognitive map; and (3) do not encode the environmental experience. Kállai et al. [23,24] asked participants to navigate through a labyrinth-like basement and to return to their starting point via the same route. First, participants completed a questionnaire measuring anxiety and drew a map of the route they followed on their return. Then, the authors collected participants’ exploration-related movements (the frequency and intensity of trunk and head rotation, touching oneself, and folding one’s arms across the chest) and physiological variables (blood pressure and heart rate) before and after the labyrinth walk. The results showed that individuals with AG exhibited a greater impairment in their spatial exploration, quantified by a higher probability of getting lost, less use of landmarks, and less accuracy in their maps or in tracing their return route. Moreover, they had higher anxiety during the task, had increased blood pressure, and exhibited disrupted exploratory activity (e.g., they turned their heads and trunk more frequently). Similarly, Jones et al. [25] observed that when individuals with AG explored stores, they focused their attention on specific environmental features unlike other shoppers. Specifically, they paid increased attention to all elements that may impede escape, such as lifts and stairways, or diminish access because these features caused them anxiety. Consistent with automatic attentional bias, Kállai et al. [22] proposed an “attention fixation training” (AFT) for alleviating panic symptoms, which consists of directing attention to the external environment, forming an environmental cognitive map, and anchoring an experience in the here and now. Kállai et al. [26] tested the efficacy of AFT and required participants with PD and AG to walk a 2.5 km route and practice the elements of AFT upon entering five panic-inducing situations: (1) walking alone in a busy street, with the examiner following 20 m behind; (2) shopping alone; (3) walking alone near a busy street with the examiner out of the client’s visual field; (4) shopping with the examiner; and (5) travelling on a bus alone. AFT produced a significant reduction in heart rate in panic-inducing situations, suggesting that attentional deficits that produce spatial disorientation can be overcome. Lastly, Gorini et al. [27] investigated spatial orientation in patients with PD and AG using a virtual adaptation of the original water maze task [28]. They found that controls rapidly learned to locate the hidden target and consistently returned to it, while patients with AG differed based on their age at the onset of the disorder. The youngest individuals, affected by AG for less time, were comparable to controls in memorizing the target location, while the oldest individuals affected by AG for a longer time had developed dysfunctional cognitive and behavioural strategies that interfered with the creation of the mental map used to find the target. Although these studies have provided some insights into the spatial memory of patients with AG, the investigation of the acquisition of spatial knowledge and the basic cognitive processes underlying navigation are still largely unexplored issues.

Our aim was to better analyse the contribution of VSWM in AG, with respect to their ability to acquire spatial information, that considers the use of both egocentric and allocentric coordinates using a computer-generated square developed by Evil Agency. Moreover, the “presence of people” was included as an experimental variable due to its role in contributing to the experience of fear in this population [29]. Although navigation has been assessed using different methods, Cimadevilla et al. [30] have demonstrated that virtual reality-based tasks might be more accurate and useful than paper and pencil tests to detect individual differences. Undoubtedly, the use of virtual reality has several advantages. It is less expensive and provides a safer alternative to real-world navigation tests, which reduces the risk of inducing panic symptoms in people with AG [31]. Although virtual environments are not completely comparable to real environments, numerous studies have supported the notion that the same behavioural and neural mechanisms are activated [32,33], thus making them fairly realistic and reliable in estimating real-world behaviour.

The main goal of this study was to explore the presence of specific difficulties in acquiring spatial information in individuals with AG. Prior studies on this issue have pointed out the difficulty of patients with PD and AG, or AG alone, in acquiring and remembering an environment. However, these studies did not explore the ability to recognize landmarks, the use of egocentric and allocentric coordinates in panic-inducing (i.e., the presence of people) or non-panic-inducing (i.e., the absence of people) situations, and the VSWM that manipulates, updates, and monitors visuo-spatial information. To our knowledge, this is the first study that has considered all these variables together in individuals with AG.

The research hypotheses are as follows:

**Hypothesis** **1** **(H1).** *VSWM hypothesis.* *We suppose that people with AG will exhibit poorer performance in both the active and passive components of VSWM than controls, which should explain the difficulty in acquiring and manipulating spatial information (VSWM hypothesis). We suppose that, in addition to the automatic attentional bias theory [22], which suggests that spatial impairment in AG is triggered only in panic-inducing situations, the VSWM deficit would affect the processing of spatial information even in non-panic-inducing situations.*

**Hypothesis** **2** **(H2).** *AG navigational difficulty.* *We predict worse performance of individuals with AG in three spatial aspects (landmark recognition, the use of egocentric coordinates, and the use of allocentric coordinates) based on a general difficulty in navigation observed in previous studies [23,24,25] in panic-inducing situations and on both the automatic attentional bias theory and the VSWM hypothesis.*

**Hypothesis** **3** **(H3).** *People vs. no-people* *scenario. Finally, based on the automatic attentional bias hypothesis [22], we expect the same performance in landmark recognition and in using egocentric/allocentric coordinates by individuals with AG and controls in a non-panic-inducing situation. In contrast, and in accordance with the VSWM hypothesis, we expect worse performance of individuals with AG than controls, even in non-panic-inducing situations.*

## 2. Materials and Methods

### 2.1. Participants

A total of 350 individuals recruited at Italian university campuses and at citizen associations through notices on social networks and bulletin boards completed two questionnaires for measuring the presence of AG: the Mobility Inventory for Agoraphobia (MIA) [34] and the Agoraphobic Cognition Questionnaire (ACQ) [35] (see Stimuli and experimental conditions section). Based on the scores on the two questionnaires for agoraphobia (MIA > 1.61, cut-off score for agoraphobia [36]; ACQ > 1.52, cut-off score for agoraphobia [35]), 53 individuals with AG (MIA, M = 1.95 ± 0.39; ACQ, M = 2.03 ± 0.57; for test–retest reliability, see Stimuli and experimental conditions section) were selected (32 females; age M = 25.67 ± 3.82 years; education M = 14.69 ± 2.12 years). Once in the laboratory, their classification was verified by an expert psychologist through an interview assessing the presence of DSM 5 criteria for agoraphobia [3]: *“Marked fear or anxiety about two or more of the following situations: using public transportation, being in open spaces, in enclosed spaces, in a crowd or outside the home alone; these situations are avoided or are endured with marked anxiety about having a panic attack. The anxiety/avoidance is persistent, and it causes significant impairment in important areas of functioning”.* The participants with AG had a mean of 0.66 panic attacks in the last 3 weeks. The psychologist also assessed whether they were in a medical treatment plan and received drugs for AG. Only individuals who had persistent symptoms of agoraphobic anxiety and/or panic attacks over several months, were not yet in a medical treatment plan and were drug-free were recruited to avoid confounding effects. However, only 3 participants (females; age M = 29.00 ± 2.64 years; education M = 16.00 ± 3.00 years) reported having had a panic attack in the last 3 weeks, so we included them in our sample and did not treat them as a different group (MIA, M = 1.80 ± 0.28; ACQ, M = 2.37 ± 0.86). The MIA and ACQ scores were not significantly different between the AG and AG with panic attack groups (MIA, F_1,51_ = 0.44, *p* = 0.50; ACQ, F_1,52_ = 1.15, *p* = 0.28). As exclusion criteria, the psychologist also verified that anxiety, phobic avoidance, and panic attacks were not better accounted for other mental disorders, general medical conditions, or drug abuse [3]. None of participants were excluded. Importantly, in our study we recruited people in the first stages of the AG to evaluate the presence of early difficulties in navigation. Then, 53 healthy volunteers without AG (WOAG) (32 females; age M = 26.94 ± 4.90 years; education M = 15.18 ± 2.93 years) who obtained lower scores than the questionnaire cut-off scores (MIA < 1.61, cut-off score [34], M = 1.21 ± 0.19; ACQ < 1.52, cut-off score [35], M = 1.19 ± 0.19), and did not have other medical or psychiatric illnesses, were compared to the AG group for a total sample of 106 participants (64 females; age M = 26.31 ± 4.42 years). The MIA and ACQ scores were significantly different between the two groups (MIA, F_1,105_ = 150.59, *p* < 0.001; ACQ, F_1,105_ = 102.50, *p* < 0.001). In order to exclude demographic differences between the two groups, we performed a series of t-test analyses for two independent samples (WOAG and AG) showing that they had a comparable age (t = −1.48, *p* = 0.14) and educational background (t = −0.98, *p* = 0.32). Considering the well-known gender differences in spatial memory [20,36], the same number of men and women was included in the WOAG and AG groups (U_1,106_ = 0.00, *p* = 0.99). Lastly, given the use of a virtual device for the experiment, the rate of computer use (t_1,106_ = −0.47, *p* = 0.63) and prior use of video games (U_1,106_ = −0.19, *p* = 0.84) between the two groups was assessed (Computer Use Questionnaire [36,37,38,39]). The present sample size was comparable to or larger than previous studies on this topic [23,24,25], and its statistical power was verified using GPower3.1. software [40] (with f2 = 0.28, α = 0.05, and power = 0.80, the sample size required for the two groups was 104 participants). Informed consent was obtained from all participants.

### 2.2. Stimuli and Experimental Conditions

#### 2.2.1. The Agoraphobic Cognitions Questionnaire (ACQ)

The Agoraphobic Cognitions Questionnaire (ACQ) [35] is composed of 14 items and comprises maladaptive thoughts concerning negative consequences of experiencing anxiety. The items concern both physiological consequences (e.g., “I am going to have a heart attack”) and behavioural consequences (e.g., “I will hurt someone” or “I am going to be crazy”). Each item was rated on a 5-point scale, ranging from “thought never occurs” (1) to “thought always occurs” (5) during experiences of anxiety. The scores could range from 14 to 70 points. The total score was computed by averaging responses across the single items. The ACQ showed good test–retest reliability after 31 days (r = 0.75, *n* = 48) and internal consistency (Cronbach’s alpha = 0.80, *n* = 72), showing a cut-off score of 1.52 for AG [35]. The high reliability of ACQ was also confirmed in the present study (Cronbach’s alpha = 0.91).

#### 2.2.2. The Mobility Inventory for Agoraphobia (MIA)

The Mobility Inventory for Agoraphobia (MIA) [34] assesses the degree of behavioural avoidance of dangerous places and situations and the frequency and severity of panic attacks on a 5-point scale: “I never avoid” (1) to “I always avoid” (5). Higher total scores reflected greater avoidance. The MIA is made up of two scales, the Avoidance Accompanied scale and Avoidance Alone scale. Each scale comprises five subsets that identify threatening places or situations. These subsets are places (12 items, e.g., elevators, supermarkets, etc.); large spaces (2 items, inside and outside spaces); means of transport (five items, e.g., car, train, etc.); driving or travelling (2 items, both on motorways); and situations (5 items accompanied, 6 items alone, plus 1 optional item indicated by participants, e.g., queuing, crossing a bridge, etc.). Moreover, individuals were asked to report the number of panic attacks during the prior 7 days and in the last 3 weeks. The MIA showed excellent internal consistency (Cronbach’s alpha = 0.95 for the Avoidance Accompanied scale and Cronbach’s alpha = 0.96 for the Avoidance Alone scale, *n* = 129; [35]). Moreover, Chambless et al. [36] reported a test–retest reliability coefficient of 0.86 for the Avoidance Accompanied scale and 0.90 for the Avoidance Alone scale over 8 days, whereas the coefficients were 0.75 and 0.89, respectively, over 31 days. The total score was computed by averaging responses across the individual items, and the cut-off was 1.61 for identifying AG [36]. The very good reliability of MIA was also confirmed in the present study (Cronbach’s alpha = 0.85).

#### 2.2.3. The Subjective Units of Distress Scale (SUDS)

The Subjective Units of Distress Scale (SUDS) [41,42] is a self-rated scale measuring the subjective intensity of distress currently experienced by an individual between “a state of absolute calmness” (0) and “the worst anxiety ever experienced” (100). It was completed by participants to verify whether our manipulation (environment less or more stressful) was effective. The SUDS was incorporated into the standard treatment protocol used in virtual reality. It is an important tool for therapists in the evaluation of treatment processes [42].

#### 2.2.4. The Computer Use Questionnaire (CUQ)

The Computer Use Questionnaire (CUQ) [38,39] allows the evaluation of the frequency and modalities of technological device use. We analysed the frequency of use of a PC (ranging from: 1, sometimes a year or less; 2, every month; 3, once a week; 4, several times a week; and 5, everyday) and video games (coding: 1, prior use; −1, no prior use) to avoid individual differences in terms of past experience and proficiency in virtual environment navigation.

#### 2.2.5. The Corsi Blocks Task (CBT)

The Corsi Blocks task (CBT) [43] measures VSWM span. Nine wooden blocks (3 × 3 cm) were affixed to a 25 × 30 cm baseboard in a standard random configuration, as in [44], and the participants were asked to mimic different sequences of blocks of increasing length (from a 2-block sequence to a maximum 9-block sequence), as shown by the experimenter. The task was administered both in the forward and backward conditions (tapping the sequence of blocks in reverse order) that engages the passive and active components of VSWM, respectively. The span was calculated as the longest sequence reproduced. Two trials per sequence of the same length were given (as in [45]). If the participant reproduced the first trial correctly, the second trial was not presented. The task performance was stopped when participants failed to reproduce two out of two sequences of a given length. The last sequence correctly repeated corresponded to the person’s forward or backward span.

#### 2.2.6. The Virtual Reality Environment

**Figure 1:**
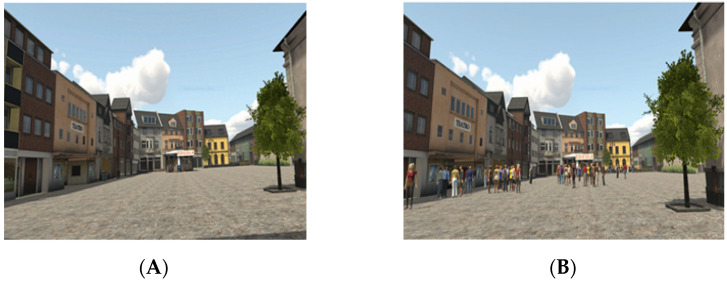
A portion of the square without people (**A**, no-people scenario) and with people blocking escape routes (**B**, people scenario).

The virtual reality environment was created by Evil Agency, and it was based on virtual environments used in previous studies [29,46]. The virtual environment represented a public square in which the following items were located: a newsstand, a fountain, a town hall, a public library, a flower shop, a museum, a theatre, a tobacco shop, a pharmacy, a bank, a jewellery store, a church, and two cafes (one with an outdoor area and the other without). The dimensions of the square were 115 × 60 metres, corresponding to the measure of a famous Italian square (Piazza Maggiore in Bologna). For study purposes, two versions of the same square were created sharing the structure including the same buildings, but one contained many people (i.e., the people scenario) standing along the entire perimeter and near the escape routes, whereas the other was without people (i.e., the no-people scenario) (Figure 1).

The virtual environment was run on a portable computer (AcerNitro 5 Notebook gaming|AN515-54) with Microsoft Windows 10 Home 64-bit, Processor Intel^®^Core™ i7-9750H Hexa-core 2.60 GHz, 39.6 cm display: 15.6” full HD with resolution 1920 × 1080 pixels, 16:9 IPS, 8 GB, DDR4 SDRAM, 1 TB HDD, 256 GB SSD, and a graphics card NVIDIA^®^GeForce^®^ GTX 1650 with 4 GB of VRAM. The environment was visualized using an immersive head mounted display (Windows Mixed Reality Headset Controller–Gyro Sensor–HDMI 2.0-USB 3.0, resolution 2.880 × 1.440 pixel, FOV 100°). Navigation and movements within the environment were possible through the use of a wireless controller.

#### 2.2.7. Spatial Tasks

**Figure 2:**
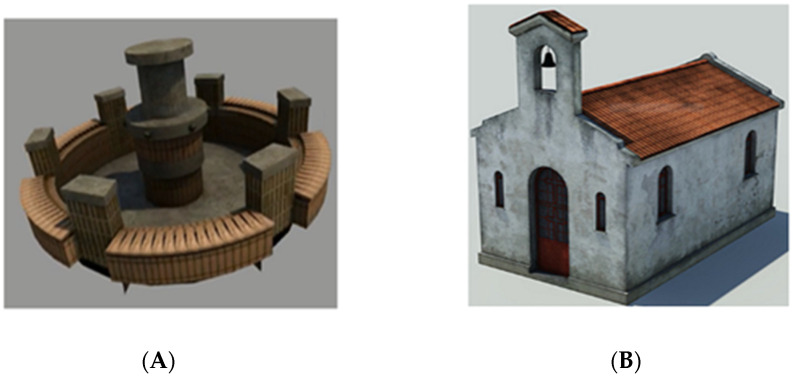
Example of target (**A**) and filler (**B**) landmarks.

The landmark recognition task (modified version of the Building task [47]) involved the participants recognizing 10 landmarks among a total of 20 buildings containing 10 targets and 10 fillers. The fillers represented landmarks of different virtual environments but were semantically congruent to be into a square (Figure 2). The score could range from 0 to 10 (if the participant correctly identified all the target elements). This task was used to evaluate landmark mental representation ability since, in order to solve it, the participants had to mentally represent perceptually salient elements without referring to any kind of spatial information.

The egocentric judgements task (a modified version of the route navigation questions [48]) explored the use of egocentric coordinates. The participants had to imagine being in a specific position and facing a particular direction within the virtual square, in order to identify a specific building located on his/her right/left/in front/behind him/her. For example, “You have the entrance of town hall in front of you. Which building is on your left?” Here, the answer was “the library”. The task included 10 questions, and the score could range from 0 to 10 (if the participant correctly answered all questions).

The allocentric judgements task [48] explored the use of allocentric coordinates. The participants had to imagine themselves at a specific point within the square and to indicate the spatial relationship between two buildings in the environment using a circular dial. The dial was used to indicate the position of 10 buildings. Half of the directional judgements were in front of the participants from the imagined perspective and half of them were behind the participants from the imagined perspective. For example, “Please, imagine having the newsstand in front of/behind you and indicate where the flower shop is in relation to the newsstand location”. The participants used 10 dials painted on sheets of papers to provide their directional judgements in a task similar to the procedure used by [49]. On the circumference of the dial, there was a mark to indicate 0°, which was the position of the first landmark (e.g., the newsstand) that the participant imagined looking towards. Successively, the participants had to place another mark on the circumference of the dial to indicate the target position (e.g., the flower shop). The score was calculated by considering the absolute angular error, corresponding to the difference in degrees between the right position of the target building and the position marked. Two means of angular errors were calculated: allocentric judgements about five pairs of buildings located in front of the participant from the imagined perspective, and five pairs of buildings located behind the participant from the imagined perspective.

### 2.3. Procedure

Participants completed the two questionnaires and a brief interview to detect agoraphobia after providing their consent. The two questionnaires and the CUQ were administered through Qualtrics software [50]. Before starting the experiment, the participants were instructed to use the virtual system equipment through a training section in a different environment from that of the study. After becoming familiarized with the system, the experiment started. The participants had to navigate for 12 min in a virtual space representing a large square (this time duration was based on [51], in which a similar virtual environment was used). They were asked to navigate while paying attention to the surrounding environment since, at the end of the navigation, they would engage in some spatial tasks about the explored environment. They had no specific constraints and could navigate in their preferred direction. During the navigation, the experimenter also collected the SUDS measure about the level of distress experienced by the participants. Before starting the virtual reality experience, to have a baseline value, and in each successive 1 m 50 s period throughout the session, the experimenter asked the participant, “How much discomfort is being felt from a state of absolute calmness (0) to the worst anxiety ever experienced (100)”. Half of the participants in both groups (WOAG and AG) were randomly assigned to the two experimental conditions characterized by the presence or absence of people within the environment. After the virtual experience, the three spatial tasks were administered in a randomized order. The experiment lasted approximately 75 min.

### 2.4. Data Analysis

Data were analysed using SPSS 23.0. Skewness and kurtosis of the data were within ±2 [52]. A multivariate ANOVA was performed in order to evaluate the effect of the independent variables (group, AG or WOAG; scenario, people or no people) on the dependent variables (landmark recognition, egocentric judgement scores, and allocentric judgement scores). Repeated measure ANOVAs were performed in order to evaluate the effect of group and scenario on allocentric judgement scores (in front of/behind the participant from the imagined perspective) and across three periods (beginning/middle/end) of SUDS measurements. Finally, a mediation analysis was performed in order to evaluate whether VSWM mediated the association between the diagnostic group and spatial task performance. Post-hoc analyses were performed with the Bonferroni test. A Bonferroni correction was applied using a significance threshold of *p* = 0.05/2 = 0.025 after correcting the *p*-level for the two ANOVAs.

## 3. Results

### 3.1. Demographics

To assess whether there were associations between demographics (age, gender, education, and frequency of PC use) and the dependent variables (VSWM span, landmark score, egocentric judgements, and allocentric judgements for buildings located in front of/behind participants from their imagined perspective), we performed a series of regression analyses. We classified gender by coding men as −1 and women as 1 [53]. Only significant (*p* < 0.025) data are reported. No predictions by gender, age, frequency of PC use, or education were revealed for each dependent variable considered, so demographic variables were not included in the following analyses.

### 3.2. Spatial Tasks (Landmark Recognition, Egocentric Judgement Scores and Allocentric Judgement Scores)

A multivariate ANOVA with group (WOAG vs. AG) and scenario (people vs. no people) as independent variables and spatial tasks scores (landmark recognition, egocentric judgements, and allocentric judgements) as dependent variables was performed.

For clarity, we stress that for landmark and egocentric judgement tasks, the higher the number of right answers was, the higher the performance. In contrast, for allocentric judgements the absolute angular error was considered (which corresponded to the difference in degrees between the right position of the target building and the position marked by the participants), and, therefore, the higher the difference between these two values was, the lower the performance.

**Figure 3:**
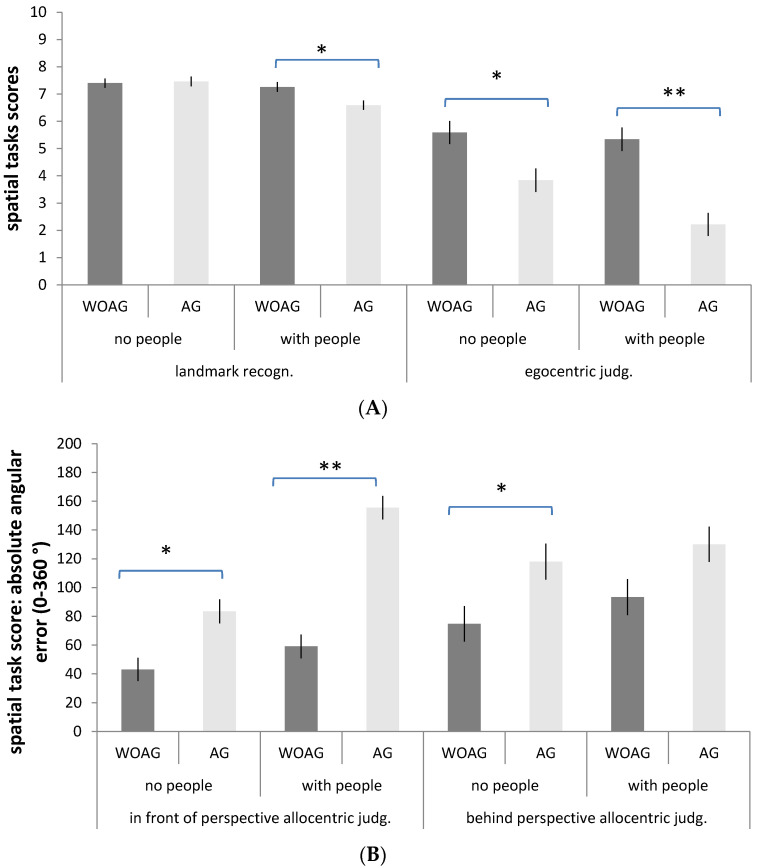
Means and standard deviations for the group x scenario interaction. (**A**) Landmark task and egocentric judgements. (**B**) Allocentric judgements. * *p* < 0.025 ** *p* < 0.001.

The analysis revealed a significant main effect of group (F_4,99_ = 24.85; *p* < 0.001, ŋ^2^ = 0.50, ŋ^2^CI = 0.37–0.57) with lower performance in the AG group than the WOAG group in all the spatial tasks (AG vs. WOAG egocentric judgements: M = 3.02, S.D. = 1.77 vs. M = 5.47, S.D. = 2.68, *p* < 0.001; AG vs. WOAG in front of participant allocentric judgements: M = 120.15, S.D. = 60.57 vs. M = 50.92, S.D. = 34.96, *p* < 0.001; AG vs. WOAG behind participant allocentric judgements: M = 124.13, S.D. = 73.75 vs. M = 83.86, S.D. = 51.82, *p* < 0.025), with the exception of the landmark measure (AG vs. WOAG landmark: M = 7.02, S.D. = 1.04 vs. M = 7.33, S.D. = 0.89; *p* = 0.08). There was a main effect of scenario (F_4,99_ = 8.78; *p* < 0.001, ŋ^2^ = 0.26, ŋ^2^CI = 0.12–0.35) with lower performance in both groups in the environment with people than in the one without people, specifically in landmark recognition and in allocentric judgements based on buildings located in front of the participant from the imagined perspective (scenario with people vs. without people: landmark, M = 6.92, S.D. = 0.97 vs. M = 7.43, S.D. = 0.92, *p* < 0.025; in front of participants allocentric judgements, M = 108.21, S.D. = 66.74 vs. M = 62.87, S.D. = 42.79, *p* < 0.001), but not in egocentric judgements (*p* = 0.03) or in allocentric judgements based on buildings located behind the participant from the imagined perspective (*p* = 0.21). Crucially, these results were qualified by a significant “group x scenario” interaction (F_4,99_ = 3.65, *p* < 0.025, ŋ^2^ = 0.12, ŋ^2^CI = 0.02–0.20). Bonferroni post-hoc comparisons showed that the two groups differed significantly in landmark recognition only in the scenario with people and showed a lower performance in the AG group than the WOAG group (*p* < 0.025), whereas there were no differences in the scenario without people (*p* = 0.83). The two groups differed significantly in egocentric judgements in both scenarios (people, *p* < 0.001; without people, *p* < 0.025); the same was revealed for allocentric judgements based on buildings located in front of the participant from the imagined perspective (people, *p* < 0.001; without people, *p* < 0.025), whereas in allocentric judgements based on buildings located behind the participant from the imagined perspective, the two groups differed significantly in the scenario without people (*p* < 0.025) but not in the one with people (*p* = 0.03). No other significant differences were revealed. Descriptive statistics in Figure 3A,B.

### 3.3. Repeated Measures ANOVA with Allocentric Judgements

To better understand the role of the task (in front of/behind participants from their imagined perspective) regarding performance on allocentric judgements, a repeated measures ANOVA was performed with group (WOAG vs. AG) and scenario (people vs. no people) as between-subject factors, task (in front of/behind participants from their imagined perspective) as a within-subject factor, and the mean of absolute angular error as a dependent variable. This revealed a main effect of group (F_1,102_ = 58.19; *p* < 0.001, ŋ^2^ = 0.36, ŋ^2^CI = 0.22–0.48) with worse performance in the AG group (M = 122.14, S.D. = 46.45) than in the WOAG group (M = 67.39, S.D. = 31.48). A main effect of scenario (F_1,102_ = 17.48 *p* < 0.001, ŋ^2^ = 0.14, ŋ^2^CI = 0.04–0.27) was also revealed, with a lower performance in the experimental scenario with people (M = 110.13, S.D. = 54.55) than in the scenario without people (M = 79.40, S.D. = 34.92). Furthermore, a main effect of the task was statistically significant (F_1,102_ = 5.83, *p* < 0.025, ŋ^2^ = 0.05, ŋ^2^CI = 0.00–0.16), showing that allocentric judgements about buildings located behind the participants from their imagined perspective (M = 103.99, S.D. = 66.58) are more difficult than judgements regarding buildings located in front of the participants from their imagined perspective (M = 85.54, S.D. = 60.26). No other significant differences were revealed.

### 3.4. VSWM Test: Forward and Backward Corsi Blocks Task

One-way ANOVA with group (WOAG and AG) as the independent variable and the mean forward VSWM span as the dependent variable showed no difference between the two groups (F_1,105_ = 3.44; *p* = 0.07, ŋ^2^ = 0.03, ŋ^2^CI = 0.00–0.11) (WOAG: M = 6.64, SD = 1.41; AG: M = 6.18, S.D. = 1.07). The same analysis considering the mean backward VSWM score showed a significant difference between the two groups (F_1,105_ = 14.08; *p* < 0.001, ŋ^2^ = 0.11, ŋ^2^CI = 0.03–0.24). The AG group (M = 5.32, S.D. = 1.13) had a smaller backward VSWM span than the WOAG group (M = 6.11, S.D. = 1.03).

### 3.5. Mediation Analysis

Previous sets of analyses have shown that the AG and WOAG groups significantly differed in both the active manipulation of visuo-spatial memory and in estimating egocentric and allocentric judgements, especially when there were people in the environment. To investigate the relation between the VSWM of participants and their performance on the spatial tasks, we performed a series of mediation analyses using Preacher–Hayes’ bootstrapping to evaluate whether VSWM mediates the association between the diagnostic group (AG vs. WOAG) and spatial task performance (landmark recognition, egocentric judgement scores, and allocentric judgement scores). Mediation analysis was carried out using Preacher–Hayes’ bootstrapping algorithm (Preacher and Hayes, 2004), measuring indirect effects by using 20,000 resamples to create a distribution of point estimates for indirect effects and test its statistical significance. The first analysis considered the group of participants and landmark scores, with VSWM as a mediator. The results indicated that VSWM did not mediate the relationship (effect = −0.08 [IC: −0.19, −0.01]) (Table 1). The second analysis considered the group of participants and egocentric scores, with VSWM as a mediator. The results indicated that VSWM did not mediate the relationship (effect = −0.21 [IC: −0.46, −0.07]) (Table 1). The third analysis considered the group of participants and allocentric judgement scores for buildings located in front of the participants from their imagined perspective, with VSWM as the mediator. The results indicated that VSWM partially mediated the effect of agoraphobia on spatial performance in this task (effect = 8.17 [IC: 3.71, 14.50]). The mediation was partial since the agoraphobic group was a significant predictor of performance in this task, even after accounting for the variance contributed by VSWM (coefficient = 26.44; SE = 4.58; t = 5.76; *p* < 0.001 [IC: 17.34, 35.53]; Z = 2.99; *p* = 0.02) (Table 1). The fourth analysis considered the group of participants and allocentric judgement scores for buildings located behind the participants from their imagined perspective, with VSWM as the mediator. The results indicated that VSWM partially mediated the effect of agoraphobia on spatial performance in this task (effect = 6.99 [IC: 2.24, 15.20]). The mediation was partial since the agoraphobic group was a significant predictor of performance in this task, even after accounting for the variance contributed by VSWM (coefficient = 13.13; SE = 6.32; t = 2.07; *p* < 0.05 [IC: 0.60, 25.67]; Z = 2.38; *p* = 0.01) (Table 1).

### 3.6. SUDS

**Figure 4:**
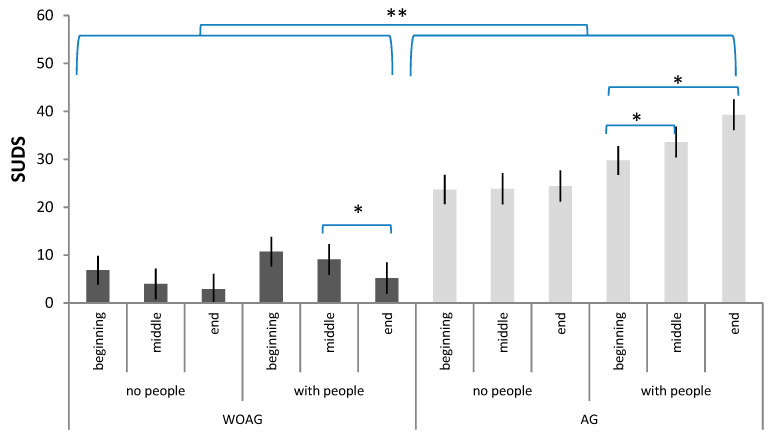
Means and standard deviations for the group x period x scenario interaction. * *p* < 0.05 ** *p* < 0.001.

Last, we considered the mean of the first three measures (beginning, 0 to 4 m 50 s), the mean of four central measures (middle, 4 m 50 s to 9 m) and the mean of the last three measures (end, 9 m to 12 m) of the SUDS collected during spatial navigation. Repeated measures ANOVA was performed with group (WOAG vs. AG) and scenario (people vs. no people) as between-subject factors, the period (beginning, middle, end) as a within-subject factor, and the mean SUDS as a dependent variable. The results revealed a main effect of group (F_1,102_ = 57.99; *p* < 0.001, ŋ^2^ = 0.36, ŋ^2^CI = 0.22−0.48) with a higher rate of SUDS in the AG group (M = 29.15, S.D. = 19.61) than in the WOAG group (M = 6.41, S.D. = 10.15). A main effect of scenario (F_1,102_ = 5.49 *p* < 0.025, ŋ^2^ = 0.05, ŋ^2^CI = 0.00−0.15) was also revealed with a higher rate of SUDS in the scenario with people (M = 21.47, S.D. = 21.84) than the scenario without people (M = 14.09, S.D. = 15.69). These results were qualified by a significant “period x group” interaction (F_1,102_ = 8.69, *p* < 0.001, ŋ^2^ = 0.14, ŋ^2^CI= 0.01−0.19). Bonferroni post-hoc comparisons showed that the AG group exhibited a higher rate of SUDS than the WOAG group at all three time points (p_s_ < 0.001). Moreover, within the WOAG group, the rate of SUDS in the beginning period (M = 8.79) was significantly higher than that in the end period (M = 4.04). In contrast, within the AG group, the rates of SUDS in the beginning period (M = 26.73) and middle period (M = 28.71) were significantly lower than those in the end period (M = 31.73). Last, a significant “period x group x scenario” interaction was revealed (F_1,102_ = 3.31, *p* < 0.05, ŋ^2^ = 0.06, ŋ^2^CI = 0.00−0.12). Bonferroni post-hoc comparisons showed that the AG group exhibited a higher rate of SUDS than the WOAG group at all three times in the scenario without people (p_s_ < 0.001) and with people (p_s_ < 0.001). Moreover, within the WOAG group, in the environment with people, the rate of SUDS in the middle (M = 9.07) was significantly higher than that in the end (M = 5.19). The opposite pattern was observed within the AG group, such that in the environment with people, the rates of SUDS in the beginning (M = 29.77) and middle (M = 33.59) were significantly lower than that in the end (M = 39.03) (Figure 4). No other significant differences were revealed.

## 4. Discussion

Our main goal was to analyse the contribution of VSWM in explaining the difficulties in acquiring and processing spatial information experienced by people with AG in order to better understand the development of clinical symptoms. Specifically, we analysed the direct relationships between VSWM abilities in the AG group and performance in tasks assessing spatial abilities (that is, the ability to recognize landmarks and use egocentric and allocentric coordinates while considering two different scenarios, i.e., with or without people). This factor has thus far been rarely considered, although it represents the most typical situation in which individuals with AG experience fear [29]. The results partially supported our hypothesis: unlike the H1-VSWM hypothesis, individuals in the AG group had a comparable forward VSWM span but a smaller backward VSWM span than those in the WOAG group. Undoubtedly, forward and backward spans reflect differences in VSWM information processing. Several studies have pointed out that the forward and backward Corsi Blocks task measure different components of VSWM [54,55,56]. The forward task is used to measure visual memory span, whereas the backward task is used to measure spatial memory span [57]. According to Logie’s model [15,58], one VSWM component is aimed at processing visual information (visual cache), and the other component processes spatial information (inner scribe). The visual cache provides temporary storage for visual information, without any kind of elaboration other than the colour and shape of objects (measured by the forward Corsi Blocks task), whereas the inner scribe handles information about movement sequences and stimuli transformation (measured by the backward Corsi Blocks task). Based on this difference, people with AG should have a lower performance in tasks that require transforming or elaborating spatial information than in tasks in which they are only required to remember visual information. Therefore, according to the H1-VSWM hypothesis, people with AG should have worse performance in both egocentric and allocentric tasks but not in the landmark recognition task, contrary to our H2-AG navigational hypothesis regarding navigational difficulties in those with AG. We found that in the landmark recognition task, the people with AG recognized fewer landmarks only in the square with people (i.e., the panic-inducing situation) in agreement with the automatic attentional bias hypothesis [22]. These individuals may acquire and process the visual characteristics of landmarks necessary to recognize the buildings in the virtual square until a phobic element is introduced. Then, their attention is captured by the latter, leading them to focus on escape routes and becoming less able to process the environment [27,59]. In agreement, Jones et al. [25] observed that individuals with AG tended to focus their attention primarily on escape routes, rather than other spatial stimuli when exploring large spaces. Importantly, here the VSWM contribution in explaining the difficulties in spatial navigation of individuals with AG was supported by their performance in allocentric judgements. In both versions of this task (in front of and behind the participants from their imagined perspective), people with AG had worse performance than those without AG, and the level of backward VSWM ability mediated the performance in this spatial task. This task requires processing environmental information and analysing the relationships between object-to-object coordinates, since the allocentric coordinates are independent from the body’s position and refer to the relations between two other spatial positions [10,60]. During navigation, individuals process allocentric environmental cues for building and retrieving topographic long-term memory. Therefore, to solve this spatial task, people have to transform and manipulate spatial information involving the inner scribe (that is, the VSWM component for processing spatial information [15]) which is disrupted in AG.

Our results confirmed deficits in solving egocentric and allocentric tasks in virtual environments with and without people in AG. On the other hand, those in the WOAG group performed better in the egocentric and allocentric tasks, which did not require a spatial perspective change (buildings located in front of the participants from the imagined perspective) than those requiring a spatial transformation (buildings located behind the participants from the imagined perspective). It is known that environmental layouts are memorized based on a preferred perspective, usually a real or imaginary viewpoint, entailing an effect called “orientation dependence” [47,61]. Finally, in the virtual square with people, regardless of the spatial task, people with AG and without AG made more errors, which was different from what was expected based on H3–People vs. no-people scenario hypothesis. This result could be explained with the “cognitive load hypothesis”, which refers to the amount of WM resources used [62]. Cognitive load theory differentiates load into three types: (i) intrinsic cognitive load, which concerns the intrinsic complexity of information that must be understood and material that must be learned; (ii) extraneous cognitive load, which concerns the manner in which instructions are provided (it may be imposed by instructions that are less than optimal, for instance non-optimal instructional procedures are considered to impose an extraneous cognitive load); and (iii) germane cognitive load, which concerns the acquisition of knowledge and refers to the learner characteristics [63]. Specifically, our environment is characterized by a different level of intrinsic cognitive load [64,65], which is the different natural complexity of information that must be processed and learned. The level of intrinsic cognitive load for a particular task and knowledge level is assumed to be determined by the level of element interactivity. Low element interactivity materials allow individual elements to be learned with minimal reference to other elements and thus impose a low working memory load, whereas high element interactivity materials consist of elements that heavily interact and so cannot be learned in isolation and impose a high working memory load. For these reasons, when facing an environment with people, individuals have to process this information in addition to the spatial elements of the environment itself, which makes the virtual square containing people much more complex to process from a cognitive perspective than the virtual square without people. The last consideration regards the level of SUDS experienced during the experiment. The people with AG perceived a higher medium level of distress than those without AG, particularly when exposed to the presence of other people. Specifically, they had a higher level of discomfort at the end of the navigation task than at the beginning and in the middle, whereas the opposite reaction was displayed by those without AG. While the WOAG group showed a habituation effect [66] to the virtual environment, the AG group showed a gradual increase in the level of discomfort during navigation, thereby boosting the feeling of fear and the need to escape from the environment [27]. However, it is important to highlight that the level of SUDS did not reach the 50% threshold, a level necessary to interrupt virtual reality exposure to avoid a participant’s panic attack [67]. 

## 5. Conclusions

From a clinical psychology perspective, our results suggest the importance of considering VSWM functioning in order to prevent or reduce agoraphobic symptoms for solving navigational problems that require the use of allocentric coordinates; VSWM could represent an early marker for the presence of AG in consideration of this mediation. Early detection of the presence of AG symptoms would improve the quality of life of these people, and, therefore, in the future it may be useful to carry out screening in order to identify in advance the presence of deficits in the active component of VSWM and to develop training to improve VSWM to reduce the onset or worsening of AG clinical symptoms. Overall, our results further define the processing abilities of people with AG and draw a stronger link between human pathologies and damage to cognitive processes. Future research could further characterize the navigational deficits in AG by collecting data on physiological indices, eye movements, and the pattern of escaping behaviour in relation to the subjective distress levels. Moreover, further research could also consider more severe stages of the disease, and compare groups of agoraphobic individuals with or without panic attacks in solving spatial tasks.

## Figures and Tables

**Table 1:** Mediation analysis was carried out using the Preacher–Hayes bootstrapping method (Preacher and Hayes, 2004).

Independent Variable	Mediator Variable	Dependent Variable	Sobel’s	Bootstrap [95% CI]
Group (1 = group with agoraphobia vs. −1 = control group)	Visuo-spatial working memory	Landmark recognition	−1.99 *	−0.08 [CI: −0.19, −0.01]
Group (1 = group with agoraphobia vs. −1 = control group)	Visuo-spatial working memory	Egocentric judgements	−2.18 *	−0.21 [CI: −0.46, −0.07]
Group (1 = group with agoraphobia vs. −1 = control group)	Visuo-spatial working memory	In front of the participant imagined perspective allocentric judgements	2.99 *	8.17 [CI: 3.71, 14.50]
Group (1 = group with agoraphobia vs. −1 = control group)	Visuo-spatial working memory	Behind the participant imagined perspective allocentric judgements	2.38 *	6.99 [CI: 2.24, 15.20]

Note: Bias-corrected and accelerated 95% CIs from 20,000 bootstrap samples are reported for specific indirect effects. * *p* < 0.05.

## Data Availability

Data are openly available in the Open Science Framework repository osf.io/t82gw.
